# Pacemaker implantation in patients with major depression, should it be of concern? A case report and literature review

**DOI:** 10.1186/s12872-020-01565-3

**Published:** 2020-06-09

**Authors:** Xiaoru Che, Youssef S. Abdelwahed, Xiaoyu Wang, Yuanjian Fang, Lihong Wang

**Affiliations:** 1 Department of Cardiology, Zhejiang Provincial People’s Hospital, People’s Hospital of Hangzhou Medical College, NO.158 Shangtang Rd, 310014 Hangzhou, People’s Republic of China; 2 Department of Cardiology, Charite´—University Medicine Berlin, Campus Benjamin Franklin, Hindenburgdamm 30, 12203 Berlin, Germany; 3 DZHK (German Centre for Cardiovascular Research), partner site Berlin, Oudenarder Str. 16, 13347 Berlin, Germany; 4 Berlin Institute of Health (BIH), Berlin, Anna-Louisa-Karsch-Straße 2, 10178 Berlin, Germany; 5 Department of Neurosurgery, The second affiliated Hospital, Scholl of Medicine, Zhejiang University, Hangzhou, China

**Keywords:** Pacemaker implantation, Depression, Psychological disease, Psychiatric disease, Suicide

## Abstract

**Background:**

Psychological adaptation after cardiac pacemaker implantation is a challenge for patients with mental illness.

**Case presentation:**

Here we report a self-harming patient with a psychiatric disorder. A 73-year-old female patient with 16-year coronary heart disease and a 4-year depression was admitted to our hospital for a coma. Two months earlier, the local hospital confirmed that the patient had a second-degree sinoatrial (SA) block (type 2) as well as basal septal hypertrophy with the left ventricular outflow obstruction. Therefore, metoprolol sustained-release tablets 95 mg QD and diltiazem sustained-release tablets 90 mg QD was given as treatment after a pacemaker was implanted. However, the patient had continued complaining about discomfort due to the pacemaker implanted after being discharged from the hospital. Two months later, she attempted to commit suicide by removing her pacemaker and taking 80 sleeping pills. After a series of treatments, the patient improved and was discharged without a pacemaker re-implantation. With continued anti-depression treatment and strengthen family supervision, the patient’s condition is stable now.

**Conclusions:**

A suicide attempt by intentionally removing the permanent pacemaker system was rarely reported. In bradycardia patients with a history of psychological or psychiatric disease, careful evaluation should be done before and after implantation of the pacemaker.

## Background

Psychological adaption after pacemaker implantation can be challenging in patients with confirmed or undetected psychiatric disorders. We represent a case of attempted suicide by the intended self-removal of a permanent pacemaker system before ingestion of 80 tablets of alprazolam in a patient with a history of major depression for four years. This case was an extreme adverse event of a depressed patient with a pacemaker. Until now, only 6 cases of severe self-harm behavior after pacemaker implantation in patients with mental diseases were reported. This case report and literature review may promote a better understanding of how to deal with a pacemaker patient with mental disease and have significant implications in clinical practice.

## Case presentation

**Fig. 1:**
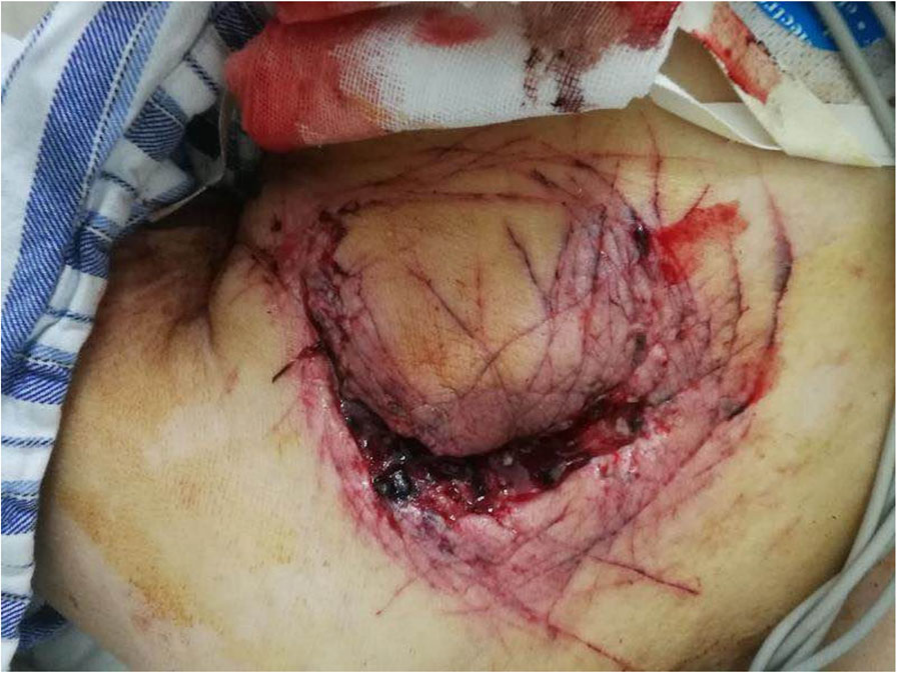
Incisional wound in the left pectoral region created by the patient

**Fig. 2:**
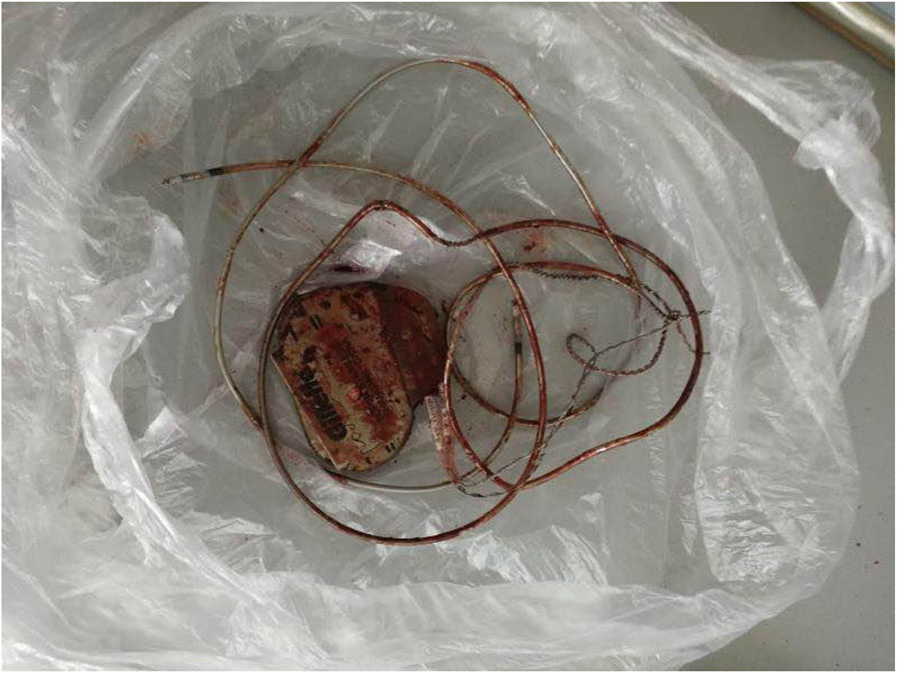
Pacemaker and electrode line extracted by the patient

A 73-year-old woman was admitted to the hospital due to a coma. Upon arrival in the emergency room (ER), the patient was found unconscious and then she was intubated due to her Glasgow Coma Scale (GCS) was 6 as well as her low blood oxygen saturation. Her other vital signs were stable. Electrocardiogram (ECG) showed sinus rhythm with heart rate at 70 bpm, and the blood pressure (BP) was around 130/70 mmHg. There was a self-inflicted wound on her left upper chest, and two silicone-caps were found in the injury. According to the patient family members, two hours before, they suddenly discovered the patient unconscious on the bedroom floor with severe trauma on her left upper chest (Fig. 1). They also found an empty packet of sleeping pills on the room floor next to the patient. Furthermore, the family detected the pacemaker and electrode lines outside the window (Fig. 2). Therefore, they assumed that the patient had removed the pacemaker and taken drugs to commit suicide. The patient’s medical history indicated that she had a history of coronary heart disease for 16 years and depression for 4 years. She had implanted two stents separately and has a long-term used aspirin, atorvastatin, and metoprolol sustained-release tablets to treat heart disease. She usually took alprazolam and paroxetine for relieving depression.

Two months earlier, the patient was admitted to another hospital due to syncope. The echocardiography was performed at that hospital showed basal septal hypertrophy and a narrow left ventricular outflow tract. Moreover, the patient underwent coronary angiography, which could exclude significant coronary artery stenosis. Intermittent left ventricular outflow tract obstruction was proven by cardiac catheterization and invasive pressure measurements. The dynamic electrocardiogram (DCG) report pointed out that the patient has a second-degree sinoatrial (SA) block (type 2). Since the patient and her family members decided not to perform septal ablation, so the patient underwent a dual-chamber pacemaker implantation due to her intermittent bradycardia during that hospitalization. The pacing generator was placed subcutaneously in the left infraclavicular region, and the two-chamber pacing leads were fixed in the right auricle and the right ventricular apex respectively through the left subclavian vein (BIOTRONIK Evia DR-T 68955978). Post-operative administration includes metoprolol sustained-release tablets 95 mg QD and diltiazem sustained-release tablets 90 mg QD treatment. Her family members mentioned that the patient had kept complaining about discomfort after she was discharged from the hospital, and she suspected her condition “had been aggravated” by the pacemaker. However, the family members did not pay any attention.

**Fig. 3:**
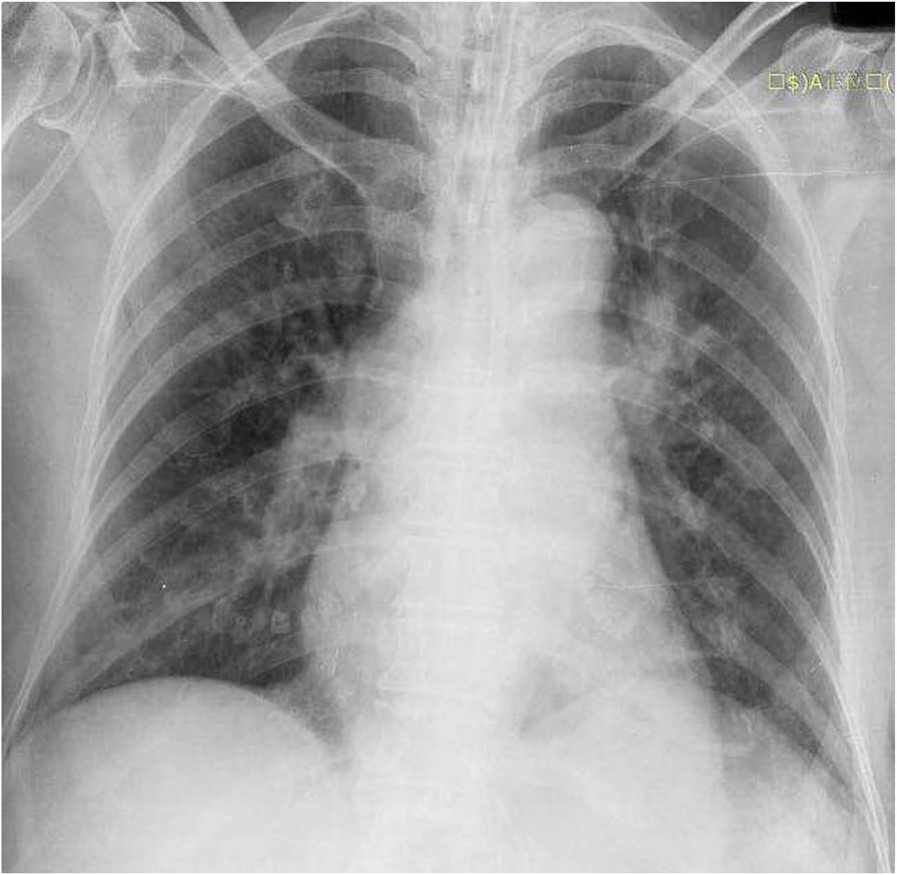
Chest x-ray demonstrating the pulse generator and the pacing leads have been removed completely

**Fig. 4:**
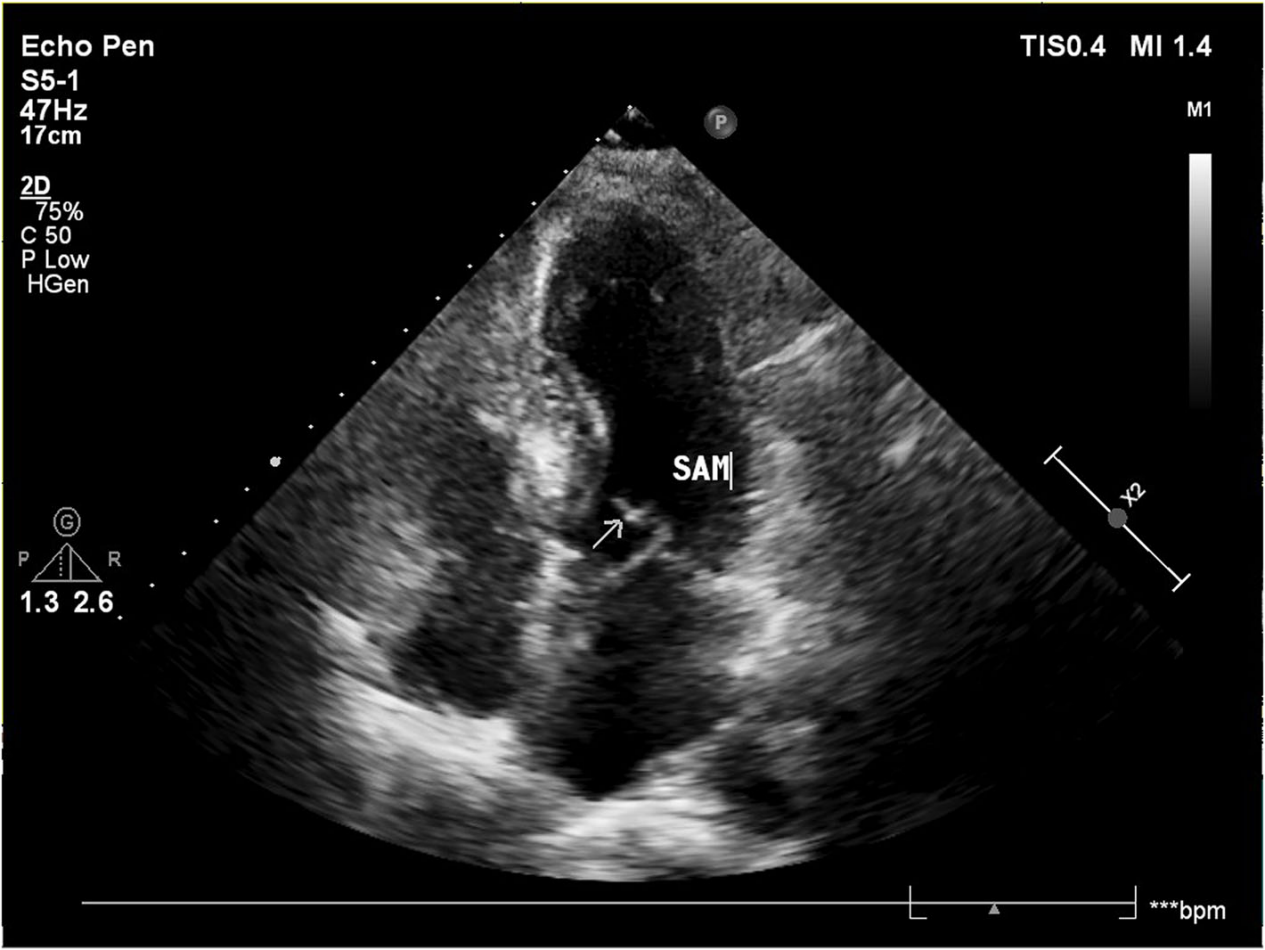
Septal hypertrophy with SAM showed in ultrasonography and no sign of pericardial effusion

In the ER, the chest radiograph indicated that the pulse generator and the pacing leads had been removed entirely (Fig. 3). There were no signs of cardiac perforation, vascular injury, or pericardial effusion by ultrasonography (Fig. 4). We performed the anti-infective treatment on the patient and surgical debridement after removed the pacemaker system residues. The electrocardiographic monitoring did not record the events of bradycardia, so a temporary pacemaker was not implanted. The patient awoke one day after. She admitted that she has gone to see the psychiatrist regularly in the hospital but has not taken any antidepressant medication prescribed by the doctor since she was last discharged from the hospital. On the day of admission to our hospital, she cut the pacemaker pocket with a knife, extracted the pacemaker by hand, and then she attempted to kill herself with 80 alprazolam tablets. As the patient and her family members refused to continue treatment in the cardiology department, they immediately left the hospital upon the improvement of the patient’s condition. Considering the particularity of this patient’s condition, the patient discharged from our hospital with aspirin, atorvastatin, and metoprolol sustained-release tablets 23.75 mg QD for her cardiac disease. To this time, no severe arrhythmias are detected during the patient’s regular visits, and no syncope symptoms occurred. With continued antidepressant treatment and strengthen family supervision, the patient’s condition is stable.

## Discussion and conclusions

Self-harm and suicidal tendencies are common in patients with mental diseases, such as depression. Self-harm is often impulsive. A terrible state of mind would be a trigger of self-harm intention [1]. This case was an extreme adverse event of a depressed patient with a pacemaker. As far as we know, severe self-harm behavior by destroying pacemaker and attempting suicide as this case had only been reported in 6 case reports (Table 1) [2–7]. In these reports, suicidal behaviors occur between 2 weeks and 12 years after pacemaker implantation, including the use of knife, gun, razor blade, and hand to destroy the pacemaker and attempt to commit suicide [2, 3, 5, 7]. One patient died of sepsis after six days, and others recovered after treatment [6].. In our case, the patient was lucky because the pacemaker was implanted recently. Thus, the pulse generator and the leads may be removed easily since the implants didn’t adhere tightly to the surrounding tissues. However, the risks of self-removal of pacemakers can be severe and fatal over time. To the best of our knowledge, the risks may include infection, chest wall injury, vascular injury, major bleeding, and even cardiac rupture. In those previous cases, the causes of suicide are all related to depression. Three patients have depression history before the pacemaker was implanted. The other three patients, although there was no established depression, the mental problems induced by their physical condition or family issues were detected and started with anti-depression therapy during their hospitalization. We also present a case with a depression history, attempted suicide after pacemaker implantation. In this case, the patient has a history of depression for four years, but her depression condition was not under control since she withdrew the anti-depression medication. While we talked with her family, we further knew that her husband slept in a separate room, and he never knew with his wife’s medication. Even, the patient had kept complaining about the pacemaker made her even worse, which meant that she could not adapt or accept the device, her family still paid no attention.

**Table 1:** The main literature of suicide after pacemaker implantation in patients with psychotic diseases

Literature	Author	Years	Ages	Gender	Pacemaker time	Pacemaker re-implantation	Psychotic history before pacemaker	Outcome
**Case 1**	Rosenthal et al .[2]	1980	81y	Male	6 m	Yes	No	Recovered
**Case 2**	Simon et al .[3]	1980	53y	Female	10y	*	Yes	Recovered
**Case 3**	Harthorne J W[4]	1980	79y	Male	11y	Yes	No	Recovered
**Case 4**	Hochmeister et al .[5]	1989	59y	Male	Not mentioned	Yes	Yes	Recovered
**Case 5**	Bordier et al .[6]	2004	70y	Male	14d	No	Not established	Died
**Case 6**	Norgaard et al. [7]	2014	70y	Male	12y	Yes	Yes	Recovered

*The previous pacemaker’s normal function was still present, not change

Psychological adaption after pacemaker implantation can be challenging in patients with confirmed or undetected psychiatric disorders [8]. From the reported literature, the early discussion focused on improving the volume and function of the pacemaker to reduce the hostility or discomfort to the patients. Nowadays, with the pacemaker optimization, the focus is more on the particular group of people with depression itself. We present another unique case that raises the issue of whether adequate evaluation and feasible criterion for pacemaker implantation should be considered in certain groups of patients? In a study by Arthur B. Simon et al., a randomized 30 patients with a cardiac pacemaker in their outpatient follow-up survey found that 20% of patients had a veiled depression or even a wish to die [3]. Młynarski R reported, 98 patients with atrioventricular blocks (AVB) showed a higher level of anxiety or depression after pacemaker implantation than preoperative by questionnaire [9]. Likewise, high levels of anxiety and depression were observed in 27.2 and 14.0%, respectively, in the 250 patients with a permanent cardiac pacemaker in Maria Polikandrioti et al.’s study [10]. All these studies showed that psychological problems such as depression have a considerable proportion of patients with pacemakers. The implanted life-saving device may involve a severe psychological burden to recipients or aggravate their symptoms. Physicians who usually pay more attention to the technical aspects of the device need to enhance awareness about psychological distress among those patients. Among patients with a pacemaker, especially in cases with a history of mental illness or suspected instances, routine follow-up, along with the systematic evaluation of anxiety or depression, and consultation with psychiatrists are necessary. Family education of depression patients is also essential.

Implantable cardioverter-defibrillator (ICD) therapy is not indicated in patients with significant psychiatric illnesses that may be aggravated by device implantation, or that may preclude systematic follow-up (Level of Evidence: C) [11, 12]. There is no question that depression should not be an absolute contraindication to permanent cardiac pacing, since chronic bradycardia may be the cause of depression and cognitive impairment, in which cases will have distinctly beneficial effects from pacemaker implantation [13]. Udo et al. have described the significant improvement of the health-related quality of life (HRQoL) in a large scale of pacemaker patients. Reason not to deny pacing to patients with mental disorders or psychiatric diseases [14]. However, a multi-disciplinary assessment for patients with mental disorders or psychiatric diseases is of great importance due to pacemaker implantation’s potential risks of deteriorating patients’ psychological conditions. It is advisable to make clear the indication to avoid unnecessary implantations. More and better post-implant surveillance in a multi-disciplinary setting should be advocated.

In conclusion, suicide attempts by intentionally removing the permanent pacemaker system are rare but have severe and adverse outcomes. The indication of pacemaker implantation should always be guided by evidence. Besides, patients and their relatives must be engaged in the decision-making process. But for these patients with mental illness, psychological or psychiatric support should be provided, a multidisciplinary assessment before and after implanting becomes essential for those patients. Even so, it is still hard to foresee the risk of self-harm or suicide in these patients; thus, the need for a feasible criterion is highlighted.

## Data Availability

The datasets used in the case are available from the corresponding author upon reasonable request.

## Abbreviations

ER: Emergency room
BP: Blood pressure
SA: Second-degree sinoatrial
ECG: Electrocardiogram
GCS: Glasgow coma scale
DCG: Dynamic electrocardiogram
